# Topic Analysis of Traditional and Social Media News Coverage of the Early COVID-19 Pandemic and Implications for Public Health Communication

**DOI:** 10.1017/dmp.2021.65

**Published:** 2021-03-03

**Authors:** Wallace Chipidza, Elmira Akbaripourdibazar, Tendai Gwanzura, Nicole M. Gatto

**Affiliations:** 1 Center for Information Systems and Technology, Claremont Graduate University, Claremont, CA, USA; 2 School of Community and Global Health, Claremont Graduate University, Claremont, CA, USA

**Keywords:** topic analysis, COVID-19, pandemic, communication, coronavirus

## Abstract

**Objective:**

To characterize and compare early coverage of coronavirus disease 2019 (COVID-19) in newspapers, television, and social media, and discuss implications for public health communication strategies that are relevant to an initial pandemic response.

**Methods:**

Latent Dirichlet allocation (LDA), an unsupervised topic modeling technique, analysis of 3271 newspaper articles, 40 cable news shows transcripts, 96,000 Twitter posts, and 1000 Reddit posts during March 4-12, 2020, a period chronologically early in the timeframe of the COVID-19 pandemic.

**Results:**

Coverage of COVID-19 clustered on topics such as epidemic, politics, and the economy, and these varied across media sources. Topics dominating news were not predominantly health-related, suggesting a limited presence of public health in news coverage in traditional and social media. Examples of misinformation were identified, particularly in social media.

**Conclusions:**

Public health entities should use communication specialists to create engaging informational content to be shared on social media sites. Public health officials should be attuned to their target audience to anticipate and prevent spread of common myths likely to exist within a population. This may help control misinformation in early stages of pandemics.

On December 31, 2019, the World Health Organization (WHO) was alerted to a series of cases of pneumonia of unknown etiology in Wuhan City, China, which were subsequently linked to a seafood and live animal market.^1^ Chinese researchers identified the cause of the disease later named coronavirus disease 2019 (COVID-19) by the WHO^1^ to be a new type of coronavirus.^2^ Between January and mid-March, 2020, COVID-19 spread from its epicenter to other Chinese cities and to over 150 countries across all continents.^3^ On March 11, the WHO declared COVID-19 a pandemic,^1^ and by April 29, confirmed cases exceeded 3 million globally, with 1/3 of these in the United States.^3^ The pandemic wrought havoc on public health and medical systems internationally, caused severe disease and death among a proportion of those infected, overwhelmed hospitals; resulted in closures of schools and cancellation of sports and entertainment events, led to travel restrictions and disruptions of daily life; and upended global financial markets.^4^

Communication of important information during emergency situations to affected populations is critical.^5,6^ Information from governments, public health, and medical entities during pandemics is vital to decision-making,^7^ taking actions to contain disease, and preventing further spread. A reliance on news media for communication is an expected and deliberate component of a pandemic response.^5^ In the United States, people seek and receive news information from numerous sources, including newspapers, radio, television, and, increasingly, social media, with a recent survey indicating that more than half of Americans in 2020 often get their news from various social media platforms.^8,9^ During outbreaks of novel infectious diseases, an understanding of the disease builds with time. Thus, initial knowledge gaps may exist among scientists and medical and public health professionals, which could contribute to the spread of misinformation and fake news in news media sources.^10^ While fake news is defined as information deliberately spread with the intent to mislead, misinformation is false information spread regardless of intent.^11^ Thus, while the idea that severe acute respiratory syndrome coronavirus 2 (SARS-CoV-2) was manufactured in a lab is an instance of fake news, the idea that garlic, lemon, and hot tea can cure COVID-19 is an example of misinformation. Both can be damaging to human health regardless of intent.

For traditional media, journalists may misreport information—from misunderstanding scientific facts, receiving wrong information, or through sensationalized reporting.^12^ For social media, users can participate semi- or fully anonymously, spreading false information without repercussions.^13^ The prevalence of misinformation could itself be a source of risk in pandemic situations,^14^ with the numerous options for news sources presenting a challenge to public health communication. Furthermore, the media may lend credibility to unproven treatments, or underreport ways to prevent disease spread. The question follows as to whether more effective strategies with news media would help achieve public health objectives related to prevention and control in pandemics such as COVID-19.

Previous research on media coverage of past pandemics has relied on qualitative methods for analysis. A content analysis of British media coverage of SARS in 2003 concluded that media tended to emphasize SARS as of Chinese origin, and convey that the superiority of Western medicine would contain its spread.^15^ During the H1N1 pandemic, corporate organizations adopted a more reassuring tone in response to the crisis than governmental organizations such as the Centers for Disease Control and Prevention (CDC) and the Department of Health and Human Services.^16^ Other researcher concluded the WHO and CDC’s response to the H1N1 pandemic enabled stigmatization.^17^ A mixed methods study of Dutch media coverage of H1N1 implicated both media and expert sources for overstating the virus threat.^18^ As the volume of information on news topics accumulating on the Internet expands, more sophisticated available methods of analyses are needed and may be used to study the COVID-19 pandemic.

The value of machine learning techniques such as Latent Dirichlet Allocation (LDA) in understanding health-related discussions on social media has been demonstrated. Data posted on social media have been used to aid disease surveillance during a 2011 German *Escherichia coli* outbreak,^19^ the 2010 US influenza epidemic,^20^ and the 2009 H1N1 pandemic.^21^ Topic analysis has been used to discover major health and disease topics of interest discussed on Twitter.^22^ The flow of information on social media originates within smaller, specific subcommunities before spreading to a wider audience online.^23^ Furthermore, social media has become widespread in society, providing a platform to people on important issues. While previous research shows the growing importance of social media in responding to emerging health crises, studies are needed to understand social media’s role in public health communication of pandemic-related information.

Beyond health crises, topic modeling has been applied to social media and traditional media coverage of various phenomena; for example, to derive the most interesting topics in a given era and time using content from historical newspapers,^24^ to conduct discourse analysis on how Muslims are portrayed on social media vis-à-vis traditional media,^25^ and to quantitatively describe differences in public opinion and mass media opinion.^26^ These studies underscore the utility of topic modeling for automating topic discovery in large data sets, which are increasingly the norm.

COVID-19 may be the most disruptive international health issue in modern times, and is dominating news media. This study explores the nature of the initial coverage of COVID-19 in traditional news and social media during the earliest weeks of the pandemic. Our objectives are, first, to characterize the nature of information first received by consumers of newspapers, television, and social media using LDA, without making any *a priori* hypotheses to discover topics associated with COVID-19 on different platforms. Our second objective is to compare topic configurations across platforms to analyze potential differences. Based on our observations, we discuss implications for communication strategies by public health entities that are relevant to an initial pandemic response.

## Methods

### Topic Modeling Using LDA

**Figure 1.**
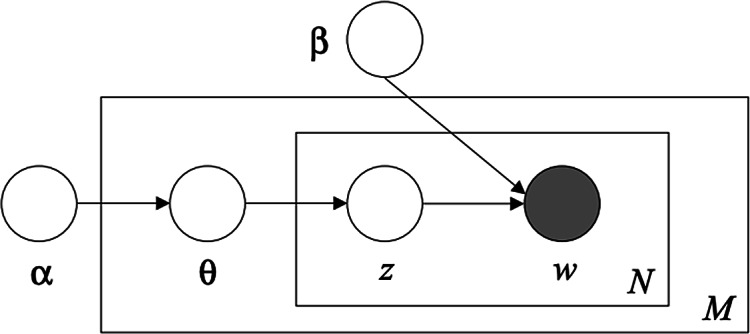
LDA in plate notation (adapted from Blei et al. 2003) with parameters: α, initialization parameter controlling the per document topic distribution; β, per topic word distribution; θ, per document topic distribution; *N*, the inner plate denoting the words contained in a given document; *M*, the outer plate denoting the documents constituting the corpus; *w*, specific word in a given document. It is the only observed variable in the model; *z*, the topic assignment for a specific word within a document.

LDA, an unsupervised machine learning technique,^27^ is an exploratory algorithm useful for discovering underlying topics within large bodies of text commonly referred to as a corpus. LDA is a generative probabilistic model in that it simulates the random process by which a given document within the corpus could have been generated.^28^ This inductive approach identifies topics that might not be anticipated. LDA has been shown to perform better than other topic modeling techniques in health-related text mining.^29^ The goal is to compute the posterior probability given evidence, that is, the conditional distribution of topics, given documents within the corpus.^28^ Calculating this requires computation of the joint probability distribution of β, θ, and *z* across all *w* (Figure 1) and dividing it by the probability of observing the corpus across all possible topic models. Algorithmically, the procedure begins with random guesses of β and θ and a prespecified number of topics, *K*. Each word in a document is randomly assigned to a topic, and this process repeats conditioned on the current topic distribution. A word is reassigned to another topic if the topic rarely appears within the document, or if the word rarely appears in the current topic. The algorithm converges when there are no new reassignments, or when the number of iterations is reached, resulting in a per document topic distribution and per topic word distribution for a corpus.

### Data Collection and Analysis

**Figure 2.**
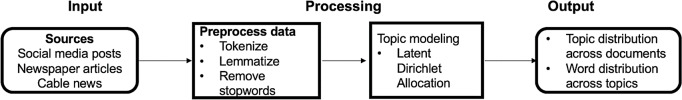
Data collection and analyses process, LDA analysis of news media.

Data from newspaper, television, and social media sources (Table 1) were obtained for March 4-12, 2020, a timeframe selected for being early in the pandemic so as to reflect initial media coverage of COVID-19. We searched keywords “coronavirus” and “COVID” in bodies of Twitter posts (by means of the Twitter application programming interface [API]), newspaper articles (by means of LexisNexis) and television transcripts (by means of NewsBank). The same keyword searches were used for titles in Reddit submissions (by means of the Reddit API), specifically the r/all subreddit, which aggregates the most popular submissions across the Reddit community. Raw data were collected and saved in text files for analyses (Figure 2). We selected Reddit and Twitter because they are influential social media platforms—they have 430 million^a^ and 330 million^b^ unique monthly visitors, respectively—and they also have APIs that permit access to and search of user content.

**Table 1.** Data sources and number of each source, words, and databases used in news media search for LDA analysis

Category	Sources	No. within each source^a^	No. of words	Databases/APIs
Newspapers	*The Associated Press*	1,588	4 million	LexisNexis
	*The New York Times*	913		
	*The Washington Post*	324		
	*Wall Street Journal*	244		
	*Los Angeles Times*	202		
Cable news shows	*Anderson Cooper 360*	4	200 thousand	NewsBank
	*All In with Chris Hayes*	6		
	*The Five*	7		
	*Hannity*	7		
	*Special Report with Bret Baier*	9		
	*Tucker Carlson Tonight*	7		
Social media	Reddit	1,000	2.5 million	Reddit API
	Twitter	96,000		Twitter API

a Number of newspaper articles, cable show episodes, Reddit posts, and Twitter tweets.

Topic analysis requires preprocessing of raw data to a specific format.^30^ We used lemmatization to convert individual words to their root words. For example, words such as, “fear”, “fears”, and “fearing” which share a common root “fear” were counted as that root. Furthermore, stopwords—words that occur frequently in the English language or the particular domain being studied—bring noise into the topic discovery process. We removed custom stopwords from the corpuses, including words such as “Monday” and “Tuesday” for newspaper articles; “crosstalk” and “Hayes” for cable news stories; “comment” and “subreddit” for Reddit posts. We used term frequency-inverse document frequency (TF-IDF) to derive the most important words for each corpus.^31^ TF-IDF is a term-weighting scheme allocating higher importance for a word appearing frequently within a document, while controlling for the word’s appearance across all documents. Thus, a word like “the” will be penalized for appearing too often within documents, meaning that it conveys limited information. We used the Python’s scikit-learn package to run LDA on the input corpora.^32^

### Understanding Topic Modeling Output

Topic modeling is an inductive approach to discovering the underlying thematic structure of a body of text. The LDA algorithm’s output is a set of topics, where a topic is defined as a cluster of co-occurring words. Thus, each topic has words that appear together more frequently than would be expected by chance.^33^ After the topics are generated, it falls on the researchers to qualitatively label them based on the content that loads with high probability on each topic. Consistent with the spirit of LDA,^28^ we prioritized interpretability of resultant topics and selected *K* = 3, to ensure adequate coverage across traditional and social media.^33^ Having retrieved the top words defining each of the topics across corpora, we scored individual components of each corpus against generated topics, and ranked them to identify which topics defined each component. Two authors (W.C., N.M.G.) derived labels based on content loading highest on individual topics, and then discussed to resolve any differences. This methodological approach is consistent with previous research using topic modeling.^33^

Institutional review board approval was not obtained as the research did not involve human subjects.

## Results

The top words defining coverage of COVID-19 across traditional and social media over the selected time period were *case, trump, travel, virus, china, world, test, health,* and *public*. Topics discovered varied based on category of media source (Table 2), and individual articles were frequently reflected in more than 1 topic. For example, an article about a football team canceling an autograph session citing fears of the virus loaded on the global topic (which included the *sports* term) in the newspaper corpus, but was also a mixture of the epidemic and economy topics.

**Table 2.** Discovered topics across traditional and social media corpora with top words in topic and top words across corpora

Source	Topic label	Top words in topic with words appearing across all corpora^a^	Representative content
Newspapers	Epidemic	healt h, virus, disease, trump, state, type, outbreak, travel, case, business, administration, home, italy	*New state and city tallies show number of people across New York infected with coronavirus rose to 105 from 89; residents prepared for workweek full of extra caution about personal contact and staying home if they notice any concerning symptoms* *(The) (c)ase of seriously ill father with deep roots in modern Orthodox community in Westchester County, NY, shows how quickly coronavirus can spread in circles that live, go to school and attend services together, with 18 people diagnosed with illness so far in community*
	Economy	public, china, industry, company, economic, market, care, infectious, presidential, word	*Market rout in stocks spilled into corporate-debt markets after investors began to more fully assess harm that prolonged economic disruption from coronavirus epidemic could do to highly indebted companies* *Oil-price war and coronavirus outbreak send investors scurrying for havens, causing 10-year Treasury yield to end day at 0.501%, having fallen as low as 0.339%, and 30-year bond to drop to 0.938% from 1.216%; long-term yields are tumbling faster than short-term yields, indicating investors expect significant slowdown*
	Global	government, sports, europe, world, test, stock, load	*Seoul expressed “extreme regret” that Japan will quarantine all visitors from South Korea due to its surging viral outbreak and warned Friday of retaliation if Tokyo doesn’t withdraw the restrictions.* *The FIFA corruption trial in the fraud case linked to the 2006 World Cup was quickly adjourned Monday with three German soccer officials absent from the courtroom near the Swiss-Italian border, close to a coronavirus outbreak.*
Cable news	Testing	test, travel, important, medical, fact, economic, pence, risk	*One you have to be clear honest and transparent about the scope of the virus and infection. And you need to test to do so. We are failing to do that miserably right now. Yesterday finally nearly a week into his tenure overseeing the response to coronavirus Vice President Mike Pence said the U.S. plans to send out 2500 testing kits by the end of the week to test up to 1.25 million people. That’s a start but it is still going to take too long to get that testing capacity up and running nationwide. And when the tests do deploy case numbers in this country will spike which will freak people out. They should be.* *I don’t think you’re calling for the testing of 320 million people. You’re saying that people who believe they may have been exposed to the virus need to know whether or not they have it and you can’t find tests for those people*
	Politics	trump, health, china, case, state, publ ic, american, world, white, doctor, fauci, government	*Just last week China’s official news service warned ominously that it might cut off drug exports to the United States to intensify the epidemic and cause even more deaths here. Keep in mind that China makes 97 percent of the world’s antibiotics. What would happen if they cut off supply to this country?* *When Coronavirus broke out in China people said this is China’s. It’s actually Donald Trump’s because there are some things you can’t lie and spin your way through above all something like a virus which is out of control*
	Consequences	virus, number, home, work, administration, disease, market, states, crisis	*In Italy which experts say is only a few weeks ahead of where we are in the development of the outbreak the entire nation is tonight in lock down. The disease has killed almost 200 people in Italy in just the past 24 hours. Markets are responding to this. The Dow dropped almost six percent today* *But what I want to talk to you about today just for a moment or two is that we would like the country to realize that as a nation we can’t be doing the kinds of things we were doing a few months ago. That it doesn’t matter if you’re in a state that has no cases or one case you have to start taking seriously what you can do now that if and when the infections will come and they will come sorry to say sad to say they will.*
Twitter	Politics	trump, breaking, positive, hanks, public, testin g, health, monkey, response, virus, travel	*Pence is LYING right now on CNN about coronavirus testing!! He just said anyone who wanted a test could get one…* *This weekend at Mar-a-Lago President Trump and Vice President Pence posed for a photo with a Brazilian government official*
	Pandemic	free, gather, italy, house, covid19, pandemic, outbreak, basketball, director, porter, barack obama, holy	*Older adults & people with a severe chronic medical condition: Prepare in advance for the possibility of a #COVID19 outbreak in your community. Take extra measures to put distance between yourself & other people to reduce your risk of being exposed.* *Italy has crossed 15k #Coronavirus cases today. Just 3 weeks ago on Feb 20th, it had 4. This is a reminder how community transmission can lead to an exponential growth and unless social distancing is implemented, this story could get repeated elsewhere.*
	Testing/ response	test, case, world, china, spread, source, overreaction	*It really strikes me that this coronavirus crisis would be a lot easier to manage if all of American society weren’t always stretched to the absolute breaking point, all day, every day. The richest country in the history of humanity, and it has no slack.* *81 countries have not reported any #COVID19 cases and 57 countries have reported 10 cases or less.*
Reddit	Epidemic	virus, test, covid, meet, china, situation, trump, risk, world, government, travel	*two weeks ago italy had twenty cases. right now there are almost 9200 cases. when it hits it hits. our hospitals are not ready for this at all.* *Masks soaked in SALT are killing the virus on contact.*
	Society	home, case, work, school, f**k, public, care, part	*I’m avoiding all public things except work.* *Rather than lament about the changes to our daily routines perhaps it would be better if we all shift our mindsets to a place of doing what’s necessary for the greater good.*
	Consequences	sick, health, number, s**t, state, life, spread, company, reason, italy, plan	*Air travel has plummeted locked out by travel bans and fear of virus-soaked airports.* *… like virtually every other disease this will disproportionately impact poorer people both internationally and here.*

a Words appearing across all corpora are indicated using underlined font.

### Newspapers

The “epidemic” topic was comprised of terms relating to the *disease outbreak* and its spread. An article in the *Wall Street Journal* that loaded on this topic reported that at least 100,000 people worldwide were infected, and estimated a case fatality rate of 2-4%. An *Associated Press* article explained how to distinguish between flu and COVID-19 symptoms. Another article loading highly on this topic from the *New York Times* advocated parents not inform their children about the virus to prevent unnecessary anxiety. In an article titled “*Italians Start Adjusting to Lockdown*,” it was revealed that the entire country of Italy was under quarantine, and this stemmed from a 36% jump in the daily death rate from COVID-19. Other articles analyzed the link between exercise and immunity, concluding that exercise bolsters immunity, although they noted that gyms may be significant factors in disease transmission.

The “economy” topic illustrated economic effects of the pandemic including reduced revenues for various companies due to lower economic activities, bankruptcy of airlines, and efforts of US legislators to increase funding. The third topic demonstrated the global impact of the pandemic with content in articles underscoring effects of COVID-19 beyond the United States. As examples, the South Korean government opposition to Japan’s decision to quarantine South Korean visitors; a France-Ireland rugby match was postponed to prevent coronavirus from spreading; and a European and Russian joint mission to launch a rover to Mars was postponed because of COVID-19-related travel restrictions.

### Cable News

The “testing” topic included coverage of the cruise ship off the California coast, and the Vice President’s announcement that passengers would be tested and quarantined if necessary. On multiple programs, cable news personalities advocated for more coronavirus testing in the United States, and South Korea was noted as an example of how to aggressively test to understand the scope of the outbreak. A commentator touted the country’s overall mitigation strategies, noting that South Korea was “*testing 20,000 people a day and they know exactly how many cases they have. And they’re busy giving them different antiviral treatments. They’re trying Plaquenil, which is a rheumatological drug. They’re trying chloroquine*…” Expedited approval of testing kits by the FDA under the emergency use provision was highlighted by some cable news hosts.

The “politics” topic included coverage highlighting 4 members of the US Congress who had self-quarantined after possible contact with COVID-19 positive individuals. Some hosts critiqued China’s actions in responding to the outbreak. One host also criticized the WHO recommendation to refer to the virus by its scientific name, insisting that it should be referred to by its place of origin. Another cable news personality compared the COVID-19 pandemic to the H1N1 pandemic of 2009/2010, noting that, while there were “*around 250 cases of the coronavirus in the US*,” H1N1 had caused “*13,000 deaths of within almost a year.*”

The third topic underscored the severe effects of the pandemic. When commentators talked about the *virus*, they also tended to talk about *home* and *work* life. Some linked the virus to disruptions of the supply chain, school closures, increased unemployment, and increased need of telemedicine and working from home. Discussions centered around the prospect of the health system being overwhelmed. Discourse also brought attention to the global economic devastation wrought by the pandemic. The possibility that the impact on the US economy would have implications on the 2020 presidential election was also highlighted.

### Twitter

Based on terms contained in the first topic, associated tweets emphasized how politics and public policy should address the pandemic. Representative tweets that loaded highly on this topic included “*Once a vaccine for coronavirus is developed it should be free.*” The second topic underscored the global nature of the crisis. Representative tweets included from the WHO: “*Of the 118000 #COVID19 cases reported globally in 114 countries more than 90 percent of cases are in just four countries*…”

The third topic contained the terms *test*, *number*, *case*, *hospital*, *battery*, and *overreaction*. It emphasized not only the need for testing, but also the backlash to the actions required to stem the spread of the pathogen. The representative tweets included “*Dear China please send us tests*,” “*Severe shortage of tests blunts coronavirus response Boston doctors say*,” “*The global pandemic of our time isn’t the Coronavirus. The global pandemic of our time is FEAR*,” and “*Holy hell people are overreacting to #coronavirus get over yourselves …*” This topic shows the major concern on the part of Twitter users regarding lack of testing capacity in the United States. It also shows a significant number of people worrying that responses by authorities amounted to an overreaction.

### Reddit

Based on terms defining the first topic, posts reflected information-seeking behavior by users wanting to know more about COVID-19. There was substantial discussion on whether fatalities varied by age; the consensus was that for young children and healthy adults, the disease was not “that dangerous.” The second topic emphasized the impact of COVID-19 on everyday life, with references to home, work, and school. Comments loading highly on this topic alerted of the possibility of school closures, working from home, and also staying indoors for an indefinite period. Other users referenced restaurants limiting their hours, staying home with children, and hoarding of supplies. The third topic articulated serious consequences of COVID-19 with references to severity of illness among patients, sports leagues in various countries canceling their seasons, and people losing jobs and incomes. Some users referenced plans for freezing mortgage and rent payments. Others were not convinced that the US government’s response to the pandemic was adequate.

### Limitations

While we intentionally selected the specific time frame for our analyses to coincide with it being early in the pandemic, a continued analysis of media coverage of the pandemic could suggest additional interpretations. This work has the benefit of hindsight such that we have now seen news topics shift multiple times since the study was undertaken. We suggest further comprehensive retrospective studies when the pandemic is over to characterize the complete trajectory of news media coverage of COVID-19, which we now know will include handling (and messaging) by 2 different US presidential administrations. Furthermore, to understand coverage on social media, we analyzed Twitter and Reddit content; future research could explore coverage on other platforms, such as Facebook and Instagram.

## Discussion

Our analysis of media sources during the initial weeks of the pandemic in the United States showed that major discovered topics included, but were not predominantly health-related, indicating less of a presence of public health, science, and medicine in early news and social media coverage of the COVID-19 pandemic compared with politics and economics. While there was substantial discussion of the presidential administration, more limited were references to ventilators, social distancing and hygiene. Absent a prominent “voice,” public health, scientific, and medical experts may have missed an opportunity to establish themselves as trustworthy and credible sources for information at the beginning of the pandemic. The period was selected for analysis because it was chronologically early in the pandemic in the United States. The 149 confirmed cases increased to 1663, and the 11 deaths increased to 40.^3^ For comparison, 10 days later on March 22, 2020, there were 33,276 confirmed cases and 417 deaths in the United States. Significant events during the period included the CDC’s changes to coronavirus testing recommendations, the WHO declaration of COVID-19 a pandemic,^34^ and the US announcement of new travel restrictions from Europe, all of which were influential to early handling of the pandemic. In retrospect, the need for an authoritative voice of health to emerge during this early period was unfilled. The minimized public health presence creates opportunities for other less informed voices to dominate, or worse, to disseminate misinformation.^6,7,35^ This was seen in cable news content where hosts downplayed the risk of infection, and on social media where users encouraged dipping masks in salt so as to “[kill] the virus on contact.” Another potentially detrimental consequence is the population not being provided with adequate educational information important to reduce spread of the virus. An analysis using structural topic modeling of a corpus of Italian online newspaper articles spanning from February to June found 3 main topics (health, economy, society) that are consistent with our results, suggesting that Western media addresses similar types of general themes in the context of the COVID-19 pandemic.^36^

From our analysis, it is quite clear that newspaper coverage of COVID-19 clustered into a few consistent themes, that is, the *Associated Press*, the *New York Times,* and the *Wall Street Journal* covered similar stories relating to rising numbers of cases in New York and turmoil in the global financial markets. On cable news and social media, on the other hand, there were more diverse interpretations. Even during the early stage in the pandemic, there is evidence that cable news hosts interpreted COVID-19 through political lenses, such as MSNBC hosts faulting the Trump administration for the lack of testing capacity in the United States, and Fox News noting the H1N1 death rate to be substantially higher than that due to COVID-19. Drugs, such as Plaquenil and chloroquine, were also being pushed as possible COVID-19 cures on cable news. Going forward, it is important for public health officials to design communication plans with knowledge and appreciation of the intended audience’s competing information.

On social media the discussions around COVID-19 were more varied. In addition to politics and the economy, social media users discussed the actor Tom Hanks’s positive diagnosis, school closures, working from home, and shortages of supplies in grocery stores. They also sought more information on how to prevent infection and possible cures. It was in response to these information-seeking activities that we observed instances of misinformation; for example, vitamin C, sodium ascorbate, and zinc were reported on Reddit as cures or preventive of COVID-19. In the absence of authoritative information from experts at the outset of a pandemic, people will seek information on social media and, unfortunately, the void will likely be filled with misinformation.

Pandemic preparedness should include communication plans that are ready and can be activated during early days of the disease’s entry and spread into a population.^6,7^ Part of preparedness could include some ready-made general informational and educational materials that could be quickly deployed, or at a minimum, templates should be available to facilitate their rapid development and publishing. For COVID-19, early indications of a novel coronavirus causing respiratory illness could have prompted public health entities to release such readied educational materials on covering coughs and sneezes. Our analysis showed instances of myths being perpetuated in social media (eg, smoking making lungs inhospitable to coronavirus). Materials that are poised for use could anticipate common myths likely to exist within a population. This should be possible if public health entities are well-acquainted with their target audience as is recommended.^6,7^

The perceived unknown and uncontrollable nature of the novel coronavirus and potential for severe disease resulting from its infection would place the pandemic in the low familiarity/high dread of Slovic’s psychometric risk paradigm^37^ or with substantial “outrage” factors following the work of Sandman^38^ serving to indicate some of the challenges^39^ underlying risk communication. The public’s perception of risk may be shaped early in a pandemic and may be difficult to change once formed.^40^ Together these emphasize the importance for public health entities to seize early communication opportunities and follow a well-conceived communication plan.^41^

In addition to the names of the president and vice president arising as top words in our analysis, we observed other proper names commonly mentioned in news sources: Hanks (for actor Tom Hanks), Porter (for US Congresswoman Katie Porter), and Barack Obama (for the former US president). One possible construal of the frequency of these names is for what they indicate the absence - the frequent mention and discussion of names of a designated spokesperson for science and health, which is considered an essential part of an effective communication plan in emergencies, and for which guidelines exist.^5-7^ In pandemic situations, perception by the public that the designated spokesperson is trustworthy and credible is essential to following instructions.^6^ It is recommended that health experts work with communication specialists to improve response activities.^7^

Given the numerous different news sources in the United States, each reaching some segment of the population, public health entities will be challenged to be “heard” within this context of competing information. To amplify the reach of messaging, public health communication during a pandemic should be deliberate in the involvement of the media.^5^ A partnership with the news media is considered a best practice.^7^ Public health, medical, and scientific entities should also recognize that the growing reliance on social media for news requires communication plans be modernized. While websites can serve as a main repository for important information and updates during a pandemic, public health entities should also establish and maintain a presence on major social media sites such as Facebook, Twitter, Instagram, YouTube, and Reddit, particularly because social media is increasingly where information is obtained by the public and circulated. An advantage to this approach is that public health entities have more control over content posted on their social media sites compared with news media sourcing information for their own coverage. Public health information could also be presented on sites in ways that will be appealing to the public. Partnerships with social media personalities adept at creating viral information could help to achieve this.

## Conclusions

Communication of important health information during times of communicable disease pandemics is crucial to informing and educating the public.^5,42^ Particularly critical for public health efforts to control an outbreak and prevent additional cases is communication during initial stages of an epidemic as disease enters a population and begins to spread. Inherent challenges exist during these initial stages as much about the agent (ie, incubation period, routes of transmission) and disease (treatment approaches, severity) may be unknown, as was the case with COVID-19 caused by the novel coronavirus. As more is learned and knowledge grows while a pandemic unfolds, it is anticipated that health entities will provide additional information to the public and correct previously disseminated information as necessary.^6,39^ Initial communication from public health officials during a novel infectious disease outbreak should acknowledge the uncertainty involved with the newly identified agent and prepare the public for situations in which additional information will be forthcoming or instructions could change over time.^39^ Modes of communication including a reliance on news and social media during a fast-moving pandemic should be nimble, flexible, and efficient for this to be achieved.^43^

## Data Availability

The data that support the findings of this study are available from the corresponding author (N.M.G.) upon reasonable request. We have shared our source code on GitHub: https://github.com/clocksian/topicanalysis.

## Footnotes

a
https://www.theverge.com/2020/12/1/21754984/reddit-dau-daily-users-revealed

b
https://www.statista.com/statistics/282087/number-of-monthly-active-twitter-users
